# Cyber Situation Comprehension for IoT Systems based on APT Alerts and Logs Correlation

**DOI:** 10.3390/s19184045

**Published:** 2019-09-19

**Authors:** Xiang Cheng, Jiale Zhang, Bing Chen

**Affiliations:** 1 College of Computer Science and Technology, Nanjing University of Aeronautics and Astronautics, Nanjing 211106, China; huozhai9527@126.com (X.C.); jlzhang@nuaa.edu.cn (J.Z.); 2 The Collaborative Innovation Center of Novel Software Technology and Industrialization, Nanjing 210023, China

**Keywords:** cyber situation comprehension, APT attack, alert correlation, log correlation, IoT, edge computing

## Abstract

With the emergence of the Advanced Persistent Threat (APT) attacks, many Internet of Things (IoT) systems have faced large numbers of potential threats with the characteristics of concealment, permeability, and pertinence. However, existing methods and technologies cannot provide comprehensive and prompt recognition of latent APT attack activities in the IoT systems. To address this problem, we propose an APT Alerts and Logs Correlation Method, named APTALCM and a framework of deploying APTALCM on the IoT system, where an edge computing architecture was used to achieve cyber situation comprehension without too much data transmission cost. Specifically, we firstly present a cyber situation ontology for modeling the concepts and properties to formalize APT attack activities in the IoT systems. Then, we introduce a cyber situation instance similarity measurement method based on the SimRank mechanism for APT alerts and logs Correlation. Combining with instance similarity, we further propose an APT alert instances correlation method to reconstruct APT attack scenarios and an APT log instances correlation method to detect log instance communities. Through the coalescence of these methods, APTALCM can accomplish the cyber situation comprehension effectively by recognizing the APT attack intentions in the IoT systems. The exhaustive experimental results demonstrate that the two kernel modules, i.e., Alert Instance Correlation Module (AICM) and Log Instance Correlation Module (LICM) in our APTALCM, can achieve both high true-positive rate and low false-positive rate.

## 1. Introduction

With the rapid advancement of the Internet of Things (IoT) infrastructure and the widely emerging networking applications, IoT security management has faced several significant challenges [1]: (a) Invisibility: it is hard to convince users to keep the software updated in IoT equipment that stops looking like traditional computers; (b) Lifetimes: since IoT devices will likely live much longer than traditional computers, unpatched software will persist much longer in IoT devices; (c) Patchability: as long-term communications were required between remote IoT devices and public networking servers, updating patches became harder than conventional information systems; (d) Consequences of Compromise: the intimate connection of IoT devices to physical infrastructure will increase the damage from successful compromise. As the above challenges, IoT systems have performed vulnerability with complexity topological structure. To cope with these increasingly complicated and potential security threats, various detection techniques have been put forward, such as the vulnerability detection technology, malicious code detection method, intrusion detection system, trying to recognize the security issues existing in the IoT systems [2,3,4,5,6]. However, the biggest shortcoming of these methods is that they cannot provide real-time recognition of the real threats from a comprehensive scope, which limits the ability of IoT security administrators to make responsive decisions. 

Recently, to solve this problem, the concept of Cyber Situation Awareness (CSA) [7] has emerged. The main idea of CSA in the large-scale IoT systems is recognizing the attack activities scattering among a large amount of noised data in IoT systems and grasping the whole IoT system security situation macroscopically. In this way, IoT system managers can make the responses appropriately and meanwhile effectively reduce the damage caused by the various attacks as possible. Among these powerful network attacks, Advanced Persistent Threat (APT) is one of the most robust multiple-steps attacks with characteristics of concealment, permeability, and pertinence, causing serious threats to all kinds of high-level information systems [3]. To mitigate the negative effects of APT, the fundamental problem is to design the cyber situation core technologies that aim at APT attack comprehension. Conventional cyber situation comprehension methods usually recognize attack intentions by only analyzing the attack alerts. However, the IoT system has the characteristics as follows: (1) the deployment of IoT terminal devices are especially scattered; (2) the majority of IoT terminal devices are resource-constraint, which means they are unable to install computing hungrily attack detecting software; (3) it is impossible to directly deploy security detection hardware for IoT terminal devices; (4) the conventional centralized cloud computing architecture cannot handle heavy transmission overheads of attack detect information. The aforementioned characteristics (1)–(3) will arise the risk of omitting alerts and failing to recognize some attack intentions. To solve this problem, we propose an APT Alerts and Logs Correlation Method (named APTALCM) to recognize the attack intentions and accomplish cyber situation comprehension in the IoT systems. To address the challenge caused by characteristic (4), we further present an edge computing-based framework for deploying APTALCM on the IoT systems, which can significantly reduce the communication overhead of cyber situation comprehension.

### 1.1. Our Contribution

In this paper, we propose APLALCM, a cyber situation comprehension method for IoT systems based on APT alerts and logs correlation. We summarize the contributions of this paper as follows:

- We propose a cyber situation comprehension method for IoT systems which can effectively, accurately and in a timely manner reconstruct the APT attack scenarios. This method is also able to dig out the potential attack activities by analyzing the log data in IoT edge devices in a holistic way, making a significant contribution to the field of IoT attack detection.
- We present a framework for deploying APTALCM on the IoT system based on edge computing architecture. This framework can greatly reduce the communication overheads of APT alerts and logs transmission among entities.
- We introduce an alert and log instances similarity measures method based on the SimRank mechanism in APTALCM. This method can provide a basis for: (1) correlating the APT alerts generated by edge servers and cloud data center; (2) correlating the logs created by edge devices.
- Experimental evaluation of the efficiency and accuracy of the proposed alert instances correlation module and log instances correlation module.

### 1.2. Organization of the Paper

The rest of the paper is organized as follows. Section 2 summarizes the background and related work of Cyber Situation Awareness. Section 3 provides an overview of cyber situation comprehension for IoT Systems, it contains a description of the proposed APTALCM and a framework of deploying APTALCM on the IoT system which is based on edge computing architecture. Section 4 presents the design details of APTALCM, which contains the cyber situation ontology construction module, alert instances correlation module, and log instances correlation module. Section 5 provides a view of our experiments and analysis. Section 6 presents the conclusions.

## 2. Background and Related Work

**Figure 1:**
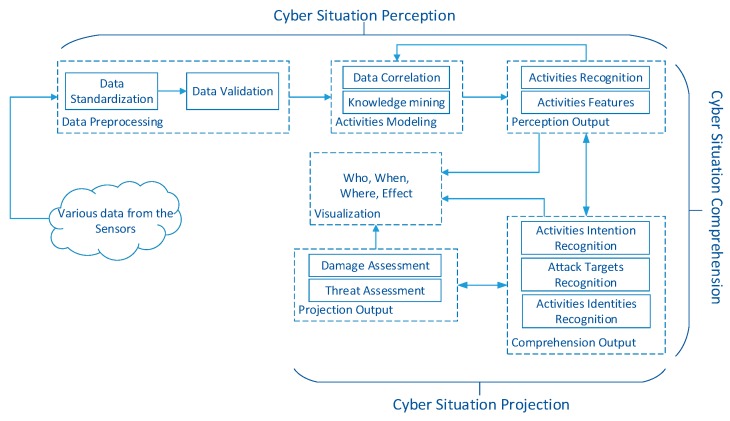
The framework of Cyber Situation Awareness.

The situation is a key factor of CSA, which means the states of various objects in the cyber systems represented by a set of measurement values. In other words, the situation is a global concept and all the objects in the cyber systems are synthesized. Any sole state cannot be regarded as a situation because they only focus on the systematic perspective and relationships between the objects in systems. Cyber situation awareness is a cognitive process applied to cyber systems consisted of three phases. First, the original data generated in the system will be fused and processed gradually to accomplish the semantics extraction of the system states and activities. Then, the recognition procedure will be executed to obtain the exiting cyberspace activities and intentions of abnormal activities in the cyberspace. At last, representational cyber situations are acquired based on the effects of recognizing activities and intentions of abnormal activities in the cyber systems. According to the definition and illustration of CSA, we can summarize the entire CSA processes into three specific operations: cyber situation perception, cyber situation comprehension, and cyber situation projection. The general functional module of CSA is shown in Figure 1 which consists of the Cyber Situation Perception module, Cyber Situation Comprehension module, Cyber Situation Projection module, and Visualization module.

- The primary duty of cyber situation perception is recognizing the activities (include attack activities) and the corresponding features in the information systems, providing for further cyber situation comprehension to acquire the attack intention.
- The cyber situation comprehension is mainly used to discover the attack activities and understand the semantics of them to acquire the attack intention.
- The cyber situation projection plays the role of analyzing and estimating the threats incurred by attack activities based on the first two phases. This kind of projection includes discovering the existing and possible effects on the objects in the cyber system incurred by attack activities. We can acquire the objective situation through projecting the cyber situation awareness results on certain objects in cyber systems. The projection process firstly constructs the cyber system situation by fusing the situation of various objects in the cyber system, then projects back the cyber situation results to further evaluate their effects.
- The cyber situation can be visualized as the formation of who on what time at where generates what impact, namely (Who, When, Where, Impact). They are, *Who* represents the recognized attack activities; *When* represents the evolutionary process of the recognized attack activities; *Where* represents the distribution of the recognized attack activities; *Impact* represents the existing effects on the cyber system incurred by the recognized attack activities. The security managers can not only observe some attack activities during a certain period time but also acquire the distribution of all the activities (contains normal activities) according to their goals and requirements.

Attack intention recognition is a part of the primary objectives of cyber situation comprehension and our work is mainly focused on the attack intention recognition of APT. Existing work [2,3,4,5,6] on attack intention recognition usually focus on attack scenarios reconstruction while ignoring the related unaggressive activities, contributing to APT attack and multiple-step attack implementation. In addition to this, the intrusion ontology used in the field of intrusion detection cannot be applied to the CSA paradigm directly. Recently, statistical analysis mechanisms are proposed to discover the relationships between attack steps. However, these methods can only perform on static databases because they all depend on expert knowledge.

At present, hot topics of cyber situation comprehension mainly focus on two branches: (1) matching the alerts with acquired attack activities based on the prior knowledge; (2) analyzing relationships between the alerts without prior knowledge. Cuppens [4] developed the LAMBDA programming to accomplish the description of templates and matching process. The researchers [5,6,7,8] usually divide an attack activity into several stages, such as the Intrusion Kill Chains Model [9]. Most recently, there are primarily two types of methods based on similarity measure: The attribute similarity method and timing sequence method. The key point of these methods is the definition of a suitable similarity measure metric. In [10,11], the authors defined a similarity function and clustered the IDS alerts based on the similarities between attributes. Later, Ourston proposed an alert correlation method [12] based on the Hidden Markov Model to get the attack sequences with the highest possibilities. Qiao proposed a simple formula for computing the similarity between alerts [13]. They apply a double clustering followed by a loose application of LCS (Longest Common Subsequence). Moreover, Murphy uses a similarity matrix based on the services each attack exploits [14,15]. Clusters of alerts are extracted using Divisive Hierarchical Clustering (DHC) on a social network graph derived from the similarity matrix. In the JEAN (Judge Evaluation of Attack Intension) system, proposed by [16], the process starts building a database of attack session graphs from a training set of IDS alerts using J-Fusion, an algorithm for alert fusion. In a brief paper, Zhang [17] presented a clustering method based on a specific metric between IP addresses.

## 3. Cyber Situation Comprehension for IoT Systems

The primary duty of cyber situation comprehension in IoT is analyzing the activities (include attack activities) and recognizing the attack intentions. Conventional cyber situation comprehension methods usually recognize attack intentions by only analyzing the attack alerts. However, the IoT system has the characteristics: (1) IoT terminal devices are deployed especially scattered; (2) the majority of IoT terminal devices have limited hardware resources which are unsuitable for installing large-scale attack detection software; (3) it is impossible to deploy security detection hardware directly on the resource-constraint IoT terminal devices. Therefore, we only can deploy the security detection equipment on the boundary of certain region IoT devices. The above characteristics will arise the risk of alerting omission and failing to recognize some attack intentions. To solve the above problems, we introduce the IoT edge device log community detection method to recognize the potential attack intentions that have not been detected; making up for the deficiencies when conventional cyber situation comprehension methods apply to the IoT systems. Then, we can acquire the activities in two forms: attack alerts and edge devices logs. As the number of activities is too large in the IoT systems, it is impossible to correlate all the activities at a short time slot. Therefore, we use the following two benchmarks to improve the efficiency of activities correlation: (1) APT attack alerts that are essential to be correlated generating the APT attack scenarios; (2) the logs generated in the edge devices infected by APT attacks are essential to be correlated to detect the unaggressive malicious activities. According to these two standpoints, results of cyber situation comprehension are composed of APT attack scenarios and log instance communities in the IoT systems. As a novelty concept, the log instance community is a kind of log instance cluster which composes of log instances that have similar attributes and operating purposes.

**Figure 2:**
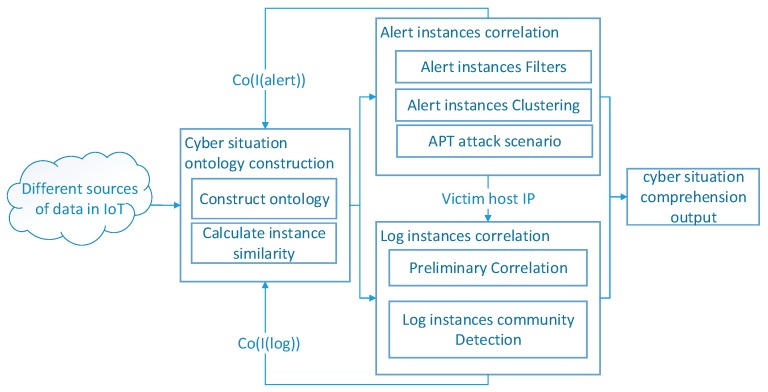
The architecture of APTALCM.

**Figure 3:**
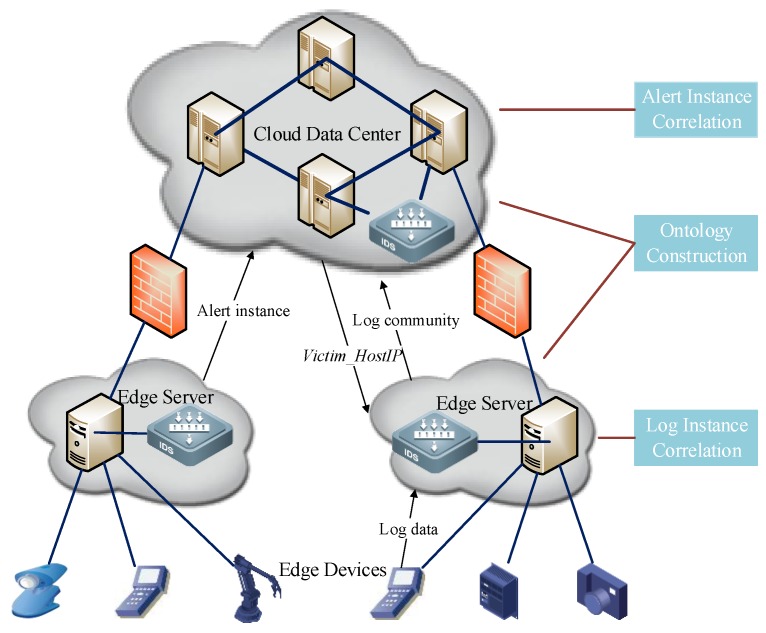
Cyber Situation Comprehension for Internet of Things (IoT) Systems Framework.

Based on the above discussions, we propose an APT Alerts and Logs Correlation Method (APTALCM) to achieve the cyber situation comprehension in the IoT systems. The architecture of APTALCM is shown in Figure 2. It needs to be emphasized that APTALCM is not only a theoretical method, but can be applied to other information systems. The large number of sensors embedded in IoT devices result in the complicated data analysis for massive attack alerts and logs. In this situation, when we apply the APTALCM based on conventional cloud computing architecture, all alerts and logs should be transmitted to a central server, so as to increase the huge communication overhead for data transmission and further affect the network performance. To address this, the emerging edge computing architecture [18] can be used to meet the challenge of data transmission costs in the IoT systems. Thus, we present an edge computing-based framework for deploying our proposed APTALCM mechanism. Figure 3 represents a typical IoT system based on edge computing architecture and it contains three layers: cloud data center, edge servers, and edge devices. Firstly, the cloud data center provides the core network access and centralized cloud computing services and management functions for IoT edge devices. Secondly, edge servers are responsible for providing virtualized and multiple management services. Finally, edge devices include all types of IoT devices (e.g., intelligent camera, automatic robot, and industrial sensing equipment) connected to the edge servers which are not only playing the role of data consumers but also data producers to participate in the distributed infrastructure for all three layers. 

For the practical implementation of our proposed APTALCM in the edge computing architecture, we describe the detail of each module of our method. Cyber situation ontology is proposed for modeling the concepts and properties of the cyber situation awareness paradigm in IoT systems. At present, there are no ontologies that are enough mature to satisfy the requirement of cyber situation awareness. Therefore, in this paper, we proposed the APTALCM, which defines a set of representational primitives for modeling the domain of cyber situation as the cyber situation ontology. The inputs of this phase are APT alerts and logs affected by APT attacks from various detection sensors in the IoT systems. The corresponding outputs are the cyber situation ontology instances. To implement the APTALCM in the system presented in Figure 3, we deploy the Ontology Construction module on cloud data center layer to convert APT alerts generated from IDS in edge servers layer, firewalls and IDS in cloud data center layer to alert instances, and we also deploy the Ontology Construction module on edge servers layer to convert the log data to log instances.

After the cyber situation ontology construction, the situation ontology instances will face two operational options according to the different instance types (alert instance & log instance). That is, alert instances raised from the preceding phase will be fed into the alert instance correlation module (AICM) and the log instances will be transmitted to the log instance correlation module (LICM). Specifically, the primary duty of AICM is to recognize APT alert instances which are belonged to an APT attack scenario in the IoT systems. To apply the APTALCM in the system presented in Figure 3, we deploy AICM on cloud data center layer to accomplish APT attack scenarios reconstruction, and then the cloud data center will transmit the *Victim_HostIp* to edge servers’ layer. Finally, the edge servers select the target IoT edge device to apply log correlation. LICM takes the log instances generated by the victim edge device as inputs to detect the log instance communities, so as to recognize the potential malicious activities. Similarly, we also deploy the LICM on the edge servers’ layer to detect log communities through the log instances and further recognize the potential malicious activities. Followed by this way, our designed systems framework has the following advantages: (1) the communication overheads between cloud data center and edge devices can be greatly reduced by deploying the edge servers layer in the middle and the shares between edge servers and cloud data center are only log community results; (2) a large part of computation costs of log community detection can be outsourced to the edge servers, so as to make up the resource-constraint drawback of IoT edge devices. Guiding by this cyber situation comprehension for IoT systems framework, the IoT system security managers can effectively acquire APT attack scenarios and log communities on cloud data center to recognize APT attack intentions in a specific IoT system based on edge computing architecture.

## 4. APTALCM Design

We have proposed the APT alerts and logs correlation method (APTALCM) to achieve the cyber situation comprehension in the IoT systems and provided the edge computing-based framework for deploying APTALCM on the IoT system. Here, we give a detailed description of our proposed APTALCM mechanism. 

### 4.1. Cyber Situation Ontology Construction 

According to the APTALCM architecture, the first module of APTALCM deployed on cloud data center and edge servers will convert the received APT alerts and edge device logs into cyber situation ontology instances. Thus, our first work is to propose a formal definition of cyber situation ontology. Different from the previously proposed methods directly send the ontology instances to attack scenarios reconstruction module, we introduce a method based on the SimRank mechanism to calculate the similarity between cyber situation instances.

#### 4.1.1. Cyber Situation Ontology Initialization

A widely accepted definition of ontology is shown as follows: the ontology defines a set of representational primitives with which to model a domain of knowledge or discourse. The representational primitives are typically classes (or concepts), attributes (or properties), and relationships (or relations between class members) [2].

According to the ontology definition and the combination of the cyber situation characteristics, we introduce a formalized definition of the cyber situation ontology as follows: O=(C, A, D, R, S). The elements **C, A, D, R** and **S** represent the set of classes (alert or log), the set of attributes, the domain of the cyber situation ontology, the set of relationships of the instances, and the set of similarity between the instances in the IoT system based on edge computing architecture (alert instances or log instances), respectively. Then, we define $A\left(c_{i}\right)$ to represent the attributes of a class $c_{i}$, $I{\left(c_{i}\right)}_{m}$ as an instance of the class $c_{i}$. Besides, the attribute value of an instance can be represented as $A\left(I{\left(c_{i}\right)}_{m}\right)$ and $\mathrm{SIM}\left(I{\left(c_{i}\right)}_{m},I{\left(c_{i}\right)}_{n}\right)$ presents the similarity between instances $I{\left(c_{i}\right)}_{m}$ and $I{\left(c_{i}\right)}_{n}$.

The APT alert class consists of alert instances converted from APT alert detected by various attack detection sensors (e.g., IDSs in edge servers, IDS in cloud data center, firewall between edge servers and cloud data center) and each alert instance represents a suspicious attack step of an APT attack in the IoT system based on edge computing architecture. In this paper, we set seven attributes for APT alert class to analyze the characteristics of APT alerts output from different attack detection sensors: *Timestamp*, *Alert_Type*, *Src_Ip, Dest_Ip*, *Src_Port*, *Dest_Port*, and *Victim_HostIp*. Attributes values of an alert instance stored in a 7-dimensional vector, A$\left(I{\left(alert\right)}_{m}\right)=\left(a_{1},a_{2},a_{3},a_{4},a_{5},a_{6},a_{7}\right)$. The description of each attribute is shown in Table 1.

The edge device log class consists of the log instances transformed from the log data generated in the edge devices. The log type depends on the operating system installed in the edge devices. On the assumption that all the edge devices are installed with Windows Embedded Compact (Windows CE), the log data that can be provided by the application programs are presented in Table 2. The essenceof transforming the log data to log instances is extracting representative attributes. In this work, we select 19 attributes from the log data are shown in Table 3. As the data generate from different application programs, not all the log instances have the same attribute type, we also give the log instance type. Attributes value of a log instance can be presented as a 19-dimensional vector: $A\left(I{\left(log\right)}_{m}\right)=\left(a_{1},a_{2}\ldots a_{18},a_{19}\right)$. If a log instance only contains attributes a1, a3, a5, the other elements of its attribute vector are zero.

#### 4.1.2. Calculate Instance Similarity

We proposed a cyber situation instance similarity calculation method to provide a correlation basis for the alert correlation module and log correlation module. Each alert or log is an instance of cyber situation ontology, and the relationship between them can be described as a labeled directed graph with similarity. As its graphic character, the proposed method is built on the SimRank mechanism.

The SimRank mechanism provides a similarity measure of structural context where the related objects are linked by directed edges. It defines a recursive function calculating the similarity between object pairs based on the concept of context. The core idea is that objects are similar in terms of referenced by similar objects.

We measure the similarity between the cyber situation instances, which fall within the same class. In other words, we measure the similarity within the alert class and edge device log class. To compare the similarity between two cyber situation instances $I{\left(c\right)}_{m}$ and $I{\left(c\right)}_{n}$ within the same class, we use the following two sets of parameters:

- Attributes: The attributes of each cyber situation instance, $A\left(I{\left(c\right)}_{m}\right)$ and $A\left(I{\left(c\right)}_{n}\right)$
- Correlated instances: The instances which have already correlated to each cyber situation instance $I{\left(c\right)}_{m}$ and $I{\left(c\right)}_{n}$ are presented as $\mathrm{Co}\left(I{\left(c\right)}_{m}\right)$ and $\mathrm{Co}\left(I{\left(c\right)}_{n}\right)$.

The basic similarity measure of cyber situation instances is calculating the similarity between their attributes and the correlated instances. The formalized representation of the similarity between two cyber situation instances is shown in Equation (1).

(1) $$\mathrm{SIM}\left(I{\left(c\right)}_{m},I{\left(c\right)}_{n}\right)=\gamma \mathrm{SIMA}\left(I{\left(c\right)}_{m},I{\left(c\right)}_{n}\right)+\beta \mathrm{SIMCo}\left(I{\left(c\right)}_{m},I{\left(c\right)}_{n}\right)\;\gamma =\frac{\left|A\left(I{\left(c\right)}_{m}\right)\cup A\left(I{\left(c\right)}_{n}\right)\right|}{\left|A\left(I{\left(c\right)}_{m}\right)\cup A\left(I{\left(c\right)}_{n}\right)\right|+\left|\mathrm{Co}\left(I{\left(c\right)}_{m}\right)\cup \mathrm{Co}\left(I{\left(c\right)}_{n}\right)\right|}\;\beta =\frac{\left|\mathrm{Co}\left(I{\left(c\right)}_{m}\right)\cup \mathrm{Co}\left(I{\left(c\right)}_{n}\right)\right|}{\left|A\left(I{\left(c\right)}_{m}\right)\cup A\left(I{\left(c\right)}_{n}\right)\right|+\left|\mathrm{Co}\left(I{\left(c\right)}_{m}\right)\cup \mathrm{Co}\left(I{\left(c\right)}_{n}\right)\right|}$$

It indicates that $\mathrm{SIMA}\left(I{\left(c\right)}_{m},I{\left(c\right)}_{n}\right)\in \left[0,1\right]$ and $\mathrm{SIMCo}\left(I{\left(c\right)}_{m},I{\left(c\right)}_{n}\right)\in \left[0,1\right]$, respectively. Two parameters γ and β are defined to normalize the impact degree where γ+β=1. Therefore, we can draw the conclusion that $\mathrm{SIM}\left(I{\left(c\right)}_{m},I{\left(c\right)}_{n}\right)\in \left[0,1\right]$. Then, we will give the formalized representation of $\mathrm{SIMA}\left(I{\left(c\right)}_{m},I{\left(c\right)}_{n}\right)$ in a mutually recursive method. $\mathrm{SIMA}\left(I{\left(c\right)}_{m},I{\left(c\right)}_{n}\right)$ measures the similarity between cyber situation instances based on attribute similarity, $\mathrm{SIMA}\left(A_{i}\left(I{\left(c\right)}_{m}\right),A_{i}\left(I{\left(c\right)}_{n}\right)\right)$ measures the similarity between the attributes of each cyber situation instance. Note that, the similarity between the same instances can be set as 1 and other conditions can be calculated in Equation (2).

(2) $$\mathrm{SIMA}\left((I{\left(c\right)}_{m},I{\left(c\right)}_{n}\right)=\frac{\partial }{\left|A\left(I\left(c\right)\right)\right|}\sum_{i=1}^{\left|A\left(I\left(c\right)\right)\right|}\mathrm{SIMA}\left(A_{i}\left(I{\left(c\right)}_{m}\right),A_{i}\left(I{\left(c\right)}_{n}\right)\right)$$

The similarity between two attributes can be set as 1 on the condition of $A_{i}\left(I{\left(c\right)}_{m}\right)=A_{i}\left(I{\left(c\right)}_{n}\right)$. If no pairs of the attributes are identical, $\mathrm{SIMA}\left(I{\left(c\right)}_{m},I{\left(c\right)}_{n}\right)$ will be calculated based on Equation (3) where ${\mathrm{Co}}_{i}\left(I{\left(c\right)}_{m}\right)$ is the correlated instance to $I{\left(c\right)}_{m}$. The method of acquiring them will be discussed in Section 4.2 and Section 4.3.

(3) $$\mathrm{SIMA}\left(I{\left(c\right)}_{m},I{\left(c\right)}_{n}\right)=\frac{\partial \sum_{i=1}^{\left|\mathrm{Co}\left(I{\left(c\right)}_{m}\right)\right|}\sum_{j=1}^{\left|\mathrm{Co}\left(I{\left(c\right)}_{n}\right)\right|}\mathrm{SIMA}\left({\mathrm{Co}}_{i}\left(I{\left(c\right)}_{m}\right),{\mathrm{Co}}_{j}\left(I{\left(c\right)}_{n}\right)\right)}{\left|\mathrm{Co}\left(I{\left(c\right)}_{m}\right)\right|\left|\mathrm{Co}\left(I{\left(c\right)}_{n}\right)\right|}$$

The $\mathrm{SIMCo}\left(I{\left(c\right)}_{m},I{\left(c\right)}_{n}\right)$ measures the similarity between cyber situation instances based on its correlated instances similarity and calculated in Equation (4). On the condition that either $I{\left(c\right)}_{m}$ or $I{\left(c\right)}_{n}$ does not have any correlated instance, we will hardly infer any similarity between them.

(4) $$\mathrm{SIMACo}\left(I{\left(c\right)}_{m},I{\left(c\right)}_{n}\right)=\{\begin{array}{c} 1\;\;\;\;\;\;I{\left(c\right)}_{m}=I{\left(c\right)}_{n}\\ \frac{\partial \sum_{i=1}^{\left|\mathrm{Co}\left(I{\left(c\right)}_{m}\right)\right|}\sum_{j=1}^{\left|\mathrm{Co}\left(I{\left(c\right)}_{n}\right)\right|}\mathrm{SIMCo}\left({\mathrm{Co}}_{i}\left(I{\left(c\right)}_{m}\right),{\mathrm{Co}}_{j}\left(I{\left(c\right)}_{n}\right)\right)}{\left|\mathrm{Co}\left(I{\left(c\right)}_{m}\right)\right|\left|\mathrm{Co}\left(I{\left(c\right)}_{n}\right)\right|}\;I{\left(c\right)}_{m}\neq I{\left(c\right)}_{n} \end{array}$$

### 4.2. Alert Instances Correlation 

As APT attack alerts are critical to be correlated generating the APT attack scenarios, we focus on deploying the Alert Instance Correlation module on cloud data center to accomplish APT attack scenarios reconstruction. The APT attack usually performs through a few steps with characteristics of persistent, targeted and aiming at the specific object. The final mission of APT is obtaining confidential data in the IoT systems. To achieve this goal, the attack process usually contains complex multistep. Alert instances which are extracted in edge servers and cloud data center belong to different APT attack step, Table 4 summarizes the matchup between APT attack scenario steps and alert instances. 

The first step (Intelligence Gathering) contains some passive process and the corresponding alerts are not readily be detected by network traffic sensors. The fourth step (Lateral Movement) is internal traffic within the edge devices while the APT alerts are detected from the inbound and outbound traffic. Based by the above facts, we only correlate the APT alert instances generated in Step 2, Step 3, Step 5 and Step 6 of an APT attack scenario.

The AICM outputs two kinds of correlated alert instance clusters: $Cluster_{full}$ and $Cluster_{sub}$. The $Cluster_{full}$ will be generated when AICM has correlated a full APT attack scenario during the correlation duration, which has every step of an APT attack scenario. To be more specific, the $Cluster_{full}$ include four alert instances, which generated within different step of a full APT attack scenario. We can correlate 9 alert instance cluster patterns based on Table 4 and the APT attack life cycle. These alert instance cluster patterns can be formalized represented as:

(5) $$Cluster_{full}=P\cap C\cap A\cap D\;P=\left[I\left(p_{1}\right)\cup I\left(p_{2}\right)\cup I\left(p_{3}\right)\right],\;C=\left[I\left(c_{1}\right)\cup I\left(c_{2}\right)\cup I\left(c_{3}\right)\right],\;A=\left[I\left(a_{1}\right)\right]\;and\;D=\left[I\left(d_{1}\right)\right].$$

The $Cluster_{sub}$ will be produced when AICM has correlated two or three rather than all steps of an APT attack scenario during the correlation duration. In this fractional correlated alert cluster, one or two step alert instances are missing. To be more specific, the $Cluster_{sub}$ includes two types of alert clusters: sub_steps_correlated_two_steps_alert_cluster and sub_steps_correlated_three_steps_ alert_cluster. We can correlate 64 alert instance cluster patterns based on Table 4 and the APT attack life cycle. These alert instance cluster patterns can be formalized represented as:

(6) $$Cluster_{sub}=\left[C\cap A\cap D\right]\cup \left[P\cap A\cap D\right]\cup \left[P\cap C\cap A\right]\cup \left[\left(P\cup C\right)\cap \left(A\cup D\right)\right]\cup \left[A\cap D\right]\cup \;\left[C\cap \left(A\cup D\right)\right]\cup \left[P\cap \left(C\cup A\cup D\right)\right]\;P=\left[I\left(p_{1}\right)\cup I\left(p_{2}\right)\cup I\left(p_{3}\right)\right],\;C=\left[I\left(c_{1}\right)\cup I\left(c_{2}\right)\cup I\left(c_{3}\right)\right],\;A=\left[I\left(a_{1}\right)\right]\;and\;D=\left[I\left(d_{1}\right)\right]$$

#### 4.2.1. Alert Instance Filter (AIF)

The APT alert instances which are constructed by the cyber situation ontology construction module are fed to the alert instance correlation module. As the ATP alerts are produced by various detection sensors (e.g., IDSs in edge servers, IDS in cloud data center, firewalls between edge servers and cloud data center), the same alert instances are given the opportunity to generate during a correlation duration. The alert instance filter (AIF) discards the repeated and redundant alert instances. It checks whether the new arriving alert instance has been constructed during the correlation duration through compare the alert instance type and instance attributes value with the previous instances. It is clear, discarding invalid alert instances can reduce the computation cost of AICM.

#### 4.2.2. Alert Instance Cluster (AIC) 

The alert instance cluster module (AIC) allocates the most similar alert instances into a certain cluster. An APT full steps scenario or sub-steps scenario can present an alert instance cluster. Each alert instance presents a disparate attack step. The AIC module gets the AIF products as input and stores the alert instances during a correlation duration. The AIC module tests the possibility of clustering as soon as a new alert instance arrives based on the APT attack scenario characteristics, so as to it is restricted by the following two rules:

- Rule 1. Alert instances, which belong to the same APT attack step, should not be allocated into the same cluster.
- Rule 2. APT attack alert instances should trigger within the correlation duration and alert instances order should in accord to the APT attack life cycle.

Formally, ${\mathrm{Timestamp}}_{p},{\;\mathrm{Timestamp}}_{c},{\;\mathrm{Timestamp}}_{a}$ and ${\mathrm{Timestamp}}_{d}$ stand for the trigger time of four alert instances respectively, which belong to four attack steps of the APT scenario. They only if comply with the following criteria that can be clustered into one cluster:

(7) $${\mathrm{Timestamp}}_{p}<{\mathrm{Timestamp}}_{c}<{\mathrm{Timestamp}}_{a}<{\mathrm{Timestamp}}_{d}$$

(8) $${\mathrm{Timestamp}}_{d}-{\mathrm{Timestamp}}_{p}<CorrelationDuration$$

All the generated alert instance clusters are presented as a directed graph and scattered alert instances are linked by directed edges based on the similarity. Then the clusters will be consumed by the correlation indexing module. Each cluster is consisted of maximum of four ordered alert instances and recorded in an instance_cluster_dataset (ICD) which is deployed on the cloud data center. 

When a new alert instance arrives in AIC module, we firstly check it belongs to which APT attack step. Based on the different alert instance type, AIC chooses different operation options. We have the conclusion: Point of entry (P) which is the second step of an APT attack scenario is the first detectable attack step. When AIC gets an alert instance $I\left(p_{i}\right)$ it generates a new cluster and allocates $I\left(p_{i}\right)$ at instance_1, which is recorded in ICD. 

When AIC gets an alert instance $I\left(c_{i}\right)$(namely: Domain_flux_instance or Ip_instance or Ssl_instance) AIC inquires the similarity degrees $\mathrm{SIM}\left(I\left(c_{i}\right),I\left(p_{i}\right)\right)$ between $I\left(c_{i}\right)$ and $I\left(p_{i}\right)$ from the cyber situation ontology construction module. $I\left(p_{i}\right)$ are the instances which are already recorded in ICD in the order *instance_1* of the existing clusters. The $c_{i}$ and $p_{i}$ are different sub-classes of class alert, then $I\left(c_{i}\right)$ and $I\left(p_{i}\right)$ can be treated as I(alert). Therefore, it is feasible to calculate the value of $\mathrm{SIM}\left(I\left(c_{i}\right),I\left(p_{i}\right)\right)$. AIC adds the $I\left(c_{i}\right)$ to the order *instance_2* of cluster which not only has the largest value of $\mathrm{SIM}\left(I\left(c_{i}\right),I\left(p_{i}\right)\right)$ but also meet the flowing two conditions in Equations (9) and (10). Then we can get the correlated instance of $I\left(c_{i}\right)$: $\mathrm{Co}\left(I\left(c_{i}\right)\right)=I\left(p_{i}\right)$, and send the $\mathrm{Co}\left(I\left(c_{i}\right)\right)$ back to the cyber situation ontology construction module for later instance similarity degree calculation. If there are no suitable alert instances $I\left(p_{i}\right)$ to be chosen, AIC will generate a new cluster and allocate $I\left(c_{i}\right)$ to *instance_2* and store it in ICD.

(9) $${\mathrm{Timestamp}}_{I\left(p_{i}\right)}<{\mathrm{Timestamp}}_{I\left(c_{i}\right)}$$

(10) $${\mathrm{Timestamp}}_{I\left(c_{i}\right)}-{\mathrm{Timestamp}}_{I\left(p_{i}\right)}<CorrelationDuration$$

When AIC get an alert instance $I\left(a_{i}\right)$(namely: Scan_instance) AIC inquires the similarity degrees $\mathrm{SIM}\left(I\left(a_{i}\right),I\left(c_{i}\right)\right)$ between $I\left(a_{i}\right)$ and $I\left(c_{i}\right)$ from the cyber situation ontology construction module. $I\left(c_{i}\right)$ are the instances which are already stored in ICD at the order instance_2 of the existing clusters. AIC adds the $I\left(a_{i}\right)$ to the order instance_3 of cluster which not only has the largest value of $\mathrm{SIM}\left(I\left(a_{i}\right),I\left(c_{i}\right)\right)$ but also meets the flowing two conditions in Equations (11) and (12). If the chosen $I\left(c_{i}\right)$ is the first instance of the cluster, the above two conditions should be changed by Equations (13) and (14). Then we can get the correlated instance of $I\left(a_{i}\right)$: $\mathrm{Co}\left(I\left(a_{i}\right)\right)=I\left(c_{i}\right)$, and send the $\mathrm{Co}\left(I\left(a_{i}\right)\right)$ back to the cyber situation ontology construction module for later instance similarity degree calculation. If there are no suitable alert instances $I\left(c_{i}\right)$ to be chosen, AIC will generate a new cluster and allocate $I\left(a_{i}\right)$ to instance_3 and store it in ICD.

(11) $${\mathrm{Timestamp}}_{I\left(p_{i}\right)}<{\mathrm{Timestamp}}_{I\left(c_{i}\right)}<{\mathrm{Timestamp}}_{I\left(a_{i}\right)}$$

(12) $${\mathrm{Timestamp}}_{I\left(a_{i}\right)}-{\mathrm{Timestamp}}_{I\left(p_{i}\right)}<CorrelationDuration$$

(13) $${\mathrm{Timestamp}}_{I\left(c_{i}\right)}<{\mathrm{Timestamp}}_{I\left(a_{i}\right)}$$

(14) $${\mathrm{Timestamp}}_{I\left(a_{i}\right)}-{\mathrm{Timestamp}}_{I\left(c_{i}\right)}<CorrelationDuration$$

When AIC get an alert instance $I\left(d_{i}\right)$(namely: Tor_intance) AIC inquires the similarity degrees $\mathrm{SIM}\left(I\left(d_{i}\right),I\left(a_{i}\right)\right)$ between $I\left(d_{i}\right)$ and $I\left(a_{i}\right)$ from the cyber situation ontology construction module. $I\left(a_{i}\right)$ are the instances which are already stored in ICD at the order *instance_3* of each existing cluster. AIC adds the $I\left(d_{i}\right)$ to the order *instance_4* of cluster which not only has the largest value of $\mathrm{SIM}\left(I\left(d_{i}\right),I\left(a_{i}\right)\right)$ but also meet the flowing two conditions in Equations (15) and (16). If the chosen $I\left(a_{i}\right)$ is the first instance of the cluster the above two conditions should be changed by Equations (17) and (18). If the chosen $I\left(a_{i}\right)$ is the second instance of the cluster the above two conditions should be changed by Equations (19) and (20). Then we can get the correlated instance of $I\left(d_{i}\right)$: $\mathrm{Co}\left(I\left(d_{i}\right)\right)=I\left(a_{i}\right)$, and send the $\mathrm{Co}\left(I\left(d_{i}\right)\right)$ back to the cyber situation ontology construction module for later instance similarity degree calculation. If there are no suitable alert instances $I\left(a_{i}\right)$ to be chosen, it means the $I\left(d_{i}\right)$ has no relationships with any clusters in the ICD, AIC will discard the $I\left(d_{i}\right)$.

(15) $${\mathrm{Timestamp}}_{I\left(p_{i}\right)}<{\mathrm{Timestamp}}_{I\left(c_{i}\right)}<{\mathrm{Timestamp}}_{I\left(a_{i}\right)}<{\mathrm{Timestamp}}_{I\left(d_{i}\right)}$$

(16) $${\mathrm{Timestamp}}_{I\left(d_{i}\right)}-{\mathrm{Timestamp}}_{I\left(p_{i}\right)}<CorrelationDuration$$

(17) $${\mathrm{Timestamp}}_{I\left(a_{i}\right)}<{\mathrm{Timestamp}}_{I\left(d_{i}\right)}$$

(18) $${\mathrm{Timestamp}}_{I\left(d_{i}\right)}-{\mathrm{Timestamp}}_{I\left(a_{i}\right)}<CorrelationDuration$$

(19) $${\mathrm{Timestamp}}_{I\left(c_{i}\right)}<{\mathrm{Timestamp}}_{I\left(a_{i}\right)}<{\mathrm{Timestamp}}_{I\left(d_{i}\right)}$$

(20) $${\mathrm{Timestamp}}_{I\left(d_{i}\right)}-{\mathrm{Timestamp}}_{I\left(c_{i}\right)}<CorrelationDuration$$

**Figure 4:**
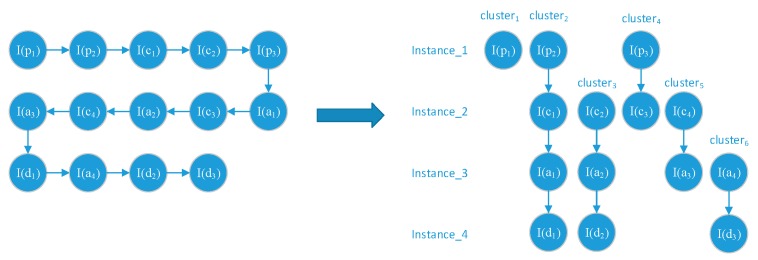
The alert instance clusters construction.

In general, we correlate the alert instance to the most similar prior-step alert instance. A correlation example is shown in Figure 4.

#### 4.2.3. APT Attack Scenario (AAS)

**Figure 5:**
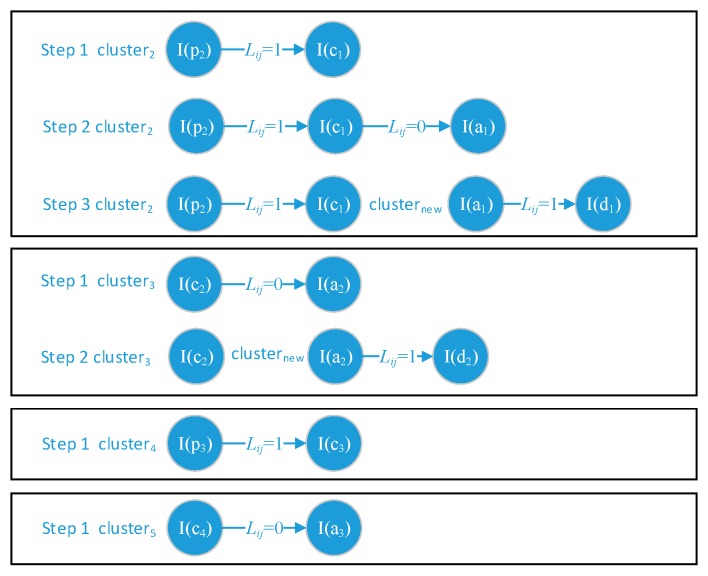
The constructing APT Attack Scenario (AAS) states evolution.

APT attack scenario (AAS) module confirms the alert instances which belonging to the same alert instance cluster whether or not can construct a full or sectional APT attack scenario in the IoT system based on edge computing architecture. As we knew each ATP alert instance cluster is constructed incrementally, it has the opportunity that later received alert instance reforms the previously correlated alert instances. To address this problem, we add a parameter $L_{ij}$ on the correlated links between every two alert instances which belong to the same cluster. The parameter $L_{ij}$ consists of two values: 1 or 0. When alert instance_i and instance_j have identical *Victim_HostIP* the $L_{ij}$ will be set as 1; otherwise, the $L_{ij}$ will be set as 0. The ASS will face four states of $L_{ij}$ during the correlation. The four states and corresponding operations are shown in Figure 5, and we describe them as follows:

- (1,1): APT alert instances can belong to a certain AAS.
- (0,1): The latest two alert instances are much more similar than the prior two alert instances. Then the first link should be disconnected and construct a new instance cluster contains the latest two alert instances waiting for the coming correlation.
- (1,0): No evidence can trigger the disconnection, just waiting for the coming instances.
- (0,0): No evidence can trigger the disconnection, just waiting for the coming instances.

We also introduce a parameter *LinkNum* to check the clustered APT alert instances and discard the uncorrelated alert instances. *LinkNum* can be formulated as follows:

(21) $$LinkNum_{cluster_{k}}=\overset{3}{\underset{\begin{array}{c} i=1,j=i+1\\ instance_{\_i}\in cluster_{k}\\ instance\_j\in cluster_{k} \end{array}}{\sum }}L_{ij}$$

LinkNum=0. APT alert instances in the cluster have no effect and cause with each other; they cannot construct an APT attack scenario.

LinkNum=1. APT alert instances in the cluster can generate a correlation between two alert instances and they can construct a $Cluster_{sub}$ with two steps.

LinkNum=2. APT alert instances in the cluster can generate correlations between three alert instances and they can construct a $Cluster_{sub}$ with three steps.

LinkNum=3. APT alert instances in the cluster can generate a correlation between four alert instances. They can construct a $Cluster_{full}$ and be presented as an APT attack scenario.

(22) $$assv_{k}=\left(cluster_{k},LinkNum,VictimHostIp,SimDeg\right)$$

(23) $$SimDee=\left(\mathrm{SIM}\left(instance_{\_1},instance_{\_2}\right),\;\mathrm{SIM}\left(instance_{\_2},instance_{\_3}\right),\mathrm{SIM}\left(instance_{\_3},instance_{\_4}\right)\right)$$

The $assv_{k}$ vectors are the sectional output of cyber situation comprehension to be used in future cyber situation projection. In the condition that $instance_{\_i}$ alert instance is absent, the value of $\mathrm{SIM}\left(instance_{\_i-1},instance_{\_i}\right)$ and $\mathrm{SIM}\left(instance_{\_i},instance_{\_i+1}\right)$ can be set as 0. Meanwhile, the LICM modules which are deployed on edge servers can get the attribute *Victim_HostIp* from the AIC module which is deployed on cloud data center to determine the correlation range of logs which are generated in edge devices.

#### 4.2.4. Module Implementation

**Figure 6:**
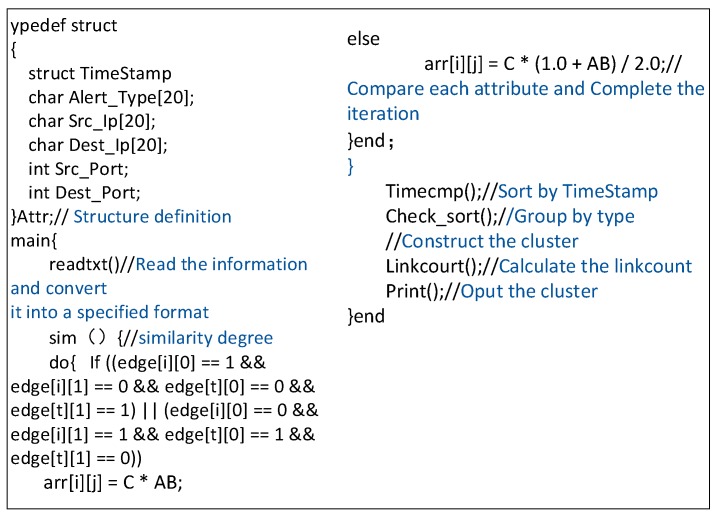
The pseudo-code of the Alert Instance Correlation Module (AICM) module.

We implement the algorithm of the AICM module in C programming language after getting the simulation dataset. The pseudo-code of the AICM module is provided in Figure 6.

### 4.3. Log Instances Correlation

Advanced Persistent Threat (APT) attack has multiple stages for the sake of being elusive and stealthy. Besides the APT alerts generated during the multiple stages, this type of attack pattern inevitably leaves some log information spatiotemporally dispersed across victim edge devices in the edge computing-based IoT systems. Therefore, we deploy the LICM on the edge servers to detect log communities through the log instances and further recognize the potential malicious activities. A large part of computation costs of log community detection can be outsourced to the edge servers, so as to make up the resource-constraint drawback of IoT edge devices. Firstly, the preliminary correlation module constructs the weighted graphs by correlating log instances which are extracted from the victim edge devices log. Then, LICM will discover the log instance communities hid within the weighted graphs by log instances community detection module.

#### 4.3.1. Preliminary Correlation (PC)

The input of preliminary correlation module (PC) are the log instances extracted from the logs which are generated in victim edge devices, the outputs are directed and weighted graphs. In this work, we represent the log instances as nodes and regard the relationships between them as edges. The weights of edges illustrate the similarity between log instances and the directions of edges illustrate the effect and cause relationship between them. Once the LICM which is deployed on an edge server gets an ASS vector from the AICM which is deployed on cloud data center it starts to construct a preliminary correlated graph based on the log instances which are extracted from the victim edge device log. Some preprocessing may leave logs before the significant APT alerts occur, so we correlate the log instances before the first alert instance generated for a short time of τ. As the log instances generated in a timing sequence, the preliminary correlation module inquires the similarity from the cyber situation ontology construction module in the same strategy. To construct a weighted and directed graph the similarity between the log instances are represented as weights according to the following strategy: 

$\left[I{\left(log\right)}_{1}\;,I{\left(log\right)}_{2},I{\left(log\right)}_{3},\ldots ,I{\left(log\right)}_{n}\right]$ is a log instance sequence generated according to the timing order. When PC module receives a new log instance $I{\left(log\right)}_{i}$ it starts to inquire the $\mathrm{SIM}\left(I{\left(log\right)}_{1},I{\left(log\right)}_{i}\right),\mathrm{SIM}\left(I{\left(log\right)}_{2},I{\left(log\right)}_{i}\right)$,$\;\mathrm{SIM}\left(I{\left(log\right)}_{3},I{\left(log\right)}_{i}\right)$, …,$\;\mathrm{SIM}\left(I{\left(log\right)}_{i-1},I{\left(log\right)}_{i}\right)$ from the cyber situation ontology construction module. On the condition that $\mathrm{SIM}\left(I{\left(log\right)}_{i},I{\left(log\right)}_{j}\right)\neq 0$, create a directed edge from $I{\left(log\right)}_{i}$ to $I{\left(log\right)}_{j}$ set $\omega_{ij}=\mathrm{SIM}\left(I{\left(log\right)}_{i},I{\left(log\right)}_{j}\right)$, generate a correlated instance of $I{\left(log\right)}_{j}$ set ${\mathrm{Co}}_{n}\left(I{\left(log\right)}_{j}\right)=I{\left(log\right)}_{i}$ and pass back ${\mathrm{Co}}_{n}\left(I{\left(log\right)}_{j}\right)$ to cyber situation ontology construction module. Otherwise, $\omega_{ij}=0$. There are no correlated relationships (edges) between $I{\left(log\right)}_{i}$ and $I{\left(log\right)}_{j}$. 

**Figure 7:**
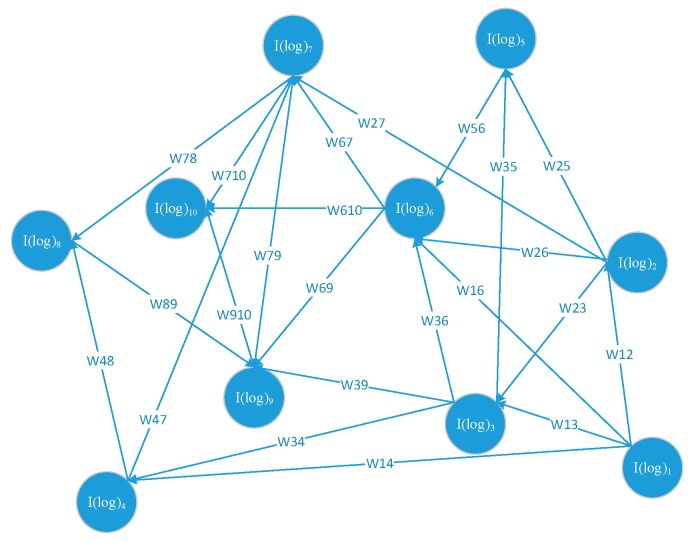
The weighted and directed log instances graph.

The PC module acquires the weighted graph of log instances is shown in Figure 7, and this module will send this graph to the log instance community detection module for later operation.

#### 4.3.2. Log Instance Community Detection (LICD) 

Taking account for the log instance graphs scale, log instance community detection module has the demand of proposing an efficient community detection method to extract the log instance communities from the intricate directed and weighted correlated instance graph. Comparing the diverse existing machine learning method used in the community detection, the LICD module owns a log instance community detection method based on the Louvain method, which has the advantage of managing large-scale nodes networks such as the edge computing-based IoT system.

At the initial phase of LICD, each log instance in the graph constructed by the PC module represents a solitary log instance community. $\sum_{m}\omega_{im}$ and $\sum_{m}\omega_{mj}$ respectively represents the totality weights added on edges associate to log instances $I{\left(log\right)}_{i}$ and $I{\left(log\right)}_{j}$, $c_{I{\left(log\right)}_{i}}$ and $c_{I{\left(log\right)}_{j}}$ respectively represents the log instance community which $I{\left(log\right)}_{i}$ and $I{\left(log\right)}_{j}$ belong to. The θ−function in the LICD module is used to separate the log instances which belong to the different log instance communities, means θ(i,j)=1 if i=j, θ(i,j)=0 otherwise. To compare the density degree of the correlations within the log instance communities with the correlations across the log instance communities, LICD introduces an evaluation index DenDeg which is defined as follows.

(24) $$DenDeg=\frac{\sum_{I{\left(log\right)}_{i},I{\left(log\right)}_{j}}\left[\omega_{ij}-\frac{\sum_{m}\omega_{im}\sum_{m}\omega_{mj}}{\sum_{I{\left(log\right)}_{i},I{\left(log\right)}_{j}}\omega_{ij}}\right]\theta \left(c_{I{\left(log\right)}_{i}},c_{I{\left(log\right)}_{j}}\right)}{\sum_{I{\left(log\right)}_{i},I{\left(log\right)}_{j}}\omega_{ij}}$$

**Figure 8:**
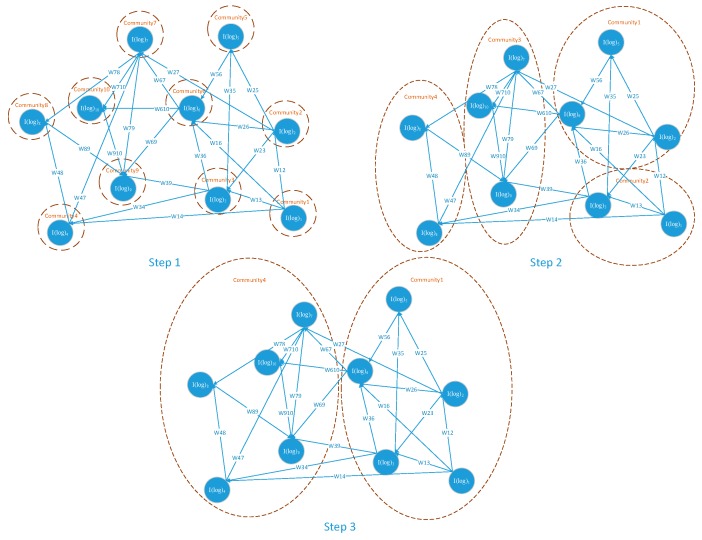
The optimization procedure of log instance communities.

As soon as the LICD module finishes the log instance initialization, it repeats actions to optimize the index DenDeg in the following strategies: for each log instance $I{\left(log\right)}_{k}$, shift $I{\left(log\right)}_{k}$ from its attached log instance community $c_{I{\left(log\right)}_{k}}$ into its correlated log instance ${\mathrm{Co}}_{n}(I{\left(log\right)}_{k})$ attached communities. LICD evaluates the value change of index DenDeg and allocates $I{\left(log\right)}_{k}$ into the log instance community $c_{I{\left(log\right)}_{k-}in}$, which has the most obvious index DenDeg increase. If the maximum increase is not positive, the log instance $I{\left(log\right)}_{k}$ will not be shifted from its original log instance community. LICD applies this process repeatedly and sequentially to each log instances until no ΔDenDeg occurs and gets the detected log instance communities as the segmental output of cyber situation comprehension. The optimization procedure of detecting log instance communities is shown in Figure 8.

#### 4.3.3. Module Implementation

**Figure 9:**
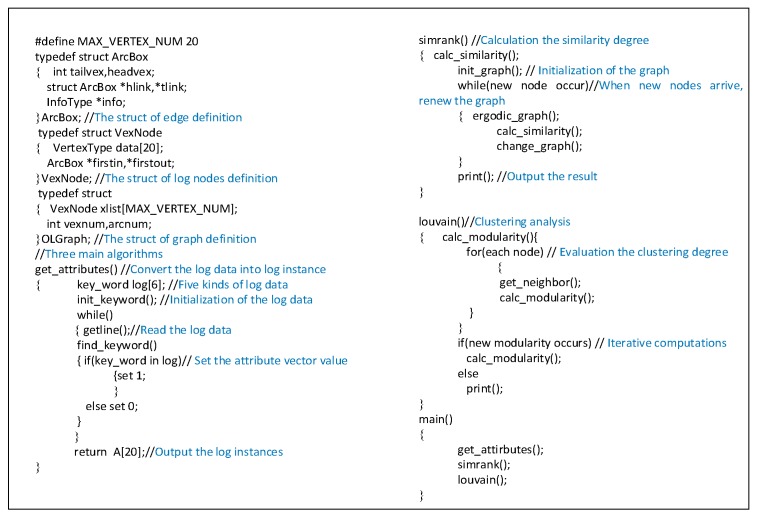
The pseudo-code of Log Instance Correlation Module (LICM) module.

We implement the algorithm of the LICM module in Python after getting the log data. The LICM module pseudo-code is provided in Figure 9:

## 5. Experimental Evaluation of APTALCM

### 5.1. Evaluation of the Alert Instance Correlation Module

As there is no available public data set that can provide enough APT attack alerts in the edge computing-based IoT system, we adapt to construct a specialized simulation data set. The duty of the alert instance correlation module is to recognize various alert instances could belong to a certain APT attack scenario. To significantly evaluate the AICM module, the simulation data set consists of APT alerts belong to APT attack scenarios and other general alerts do not belong to the APT attack scenarios. The experiment aims to verify whether the AICM module can reconstruct the APT scenarios hidden in the constructed data set. 

#### 5.1.1. Data Generation

To construct the simulation data set, we use Python to write a script, which constructs two classes of alert: Correlative alerts are part of a $Cluster_{full}$ or $Cluster_{sub}$; scattered alerts do not belong to any of the alert instance cluster. In our experiments, we set seven attributes to each alert: *Alert_Type*, *Timestamp*, *Src_Ip*, *Dest_Ip*, *Src_Port*, *Dest_Port,* and *Victim_HostIp*. To guarantee the randomness of the generated alert, we select the *Alert_Type* from the provided eight ATP alert types. We assign a random value start from 01 February 2019 00:00:01 to 30 March 2019 23:59:59 to *Timestamp*. The *Src_Ip* value is assigned based on the selected *Alert_Type*.

The *Dest_Ip* assigned randomly with an IP address in an industry IoT network. We select the *Src_Port* randomly from the 49,140 to 65,521 ranges, which are usually allocated dynamically to initiate a connection. Then, we further assign a random port number to *Dest_Port* based on the *Alert_Type*. The *Victim_HostIp* is assigned randomly with an IP address in an industry IoT network. Besides, we have generated 5000 APT alerts for the simulation data set consisted of 150 $Cluster_{full}$, 150 $Cluster_{sub}$, and 4000 random isolated alerts. In this way, the simulation data set for evaluating the AICM module have accomplished. Note that, as there is no standard APT attack data set for testing the performance of our method, we use the above constructed simulate data set to present the real APT attack in the edge computing-based IoT system. The attribute types of simulation alert data and the real APT alert are identical. The biggest difference between the simulation data set and the real APT alert is that attribute value distribution perhaps contains much more random features in the simulation data set, but the real APT alerts maybe generate follow some certain intentions guided by attackers. However, it will not limit to disclose the correlated ability of our method if the data set is scattered.

#### 5.1.2. Correlation Performance

After the data generation phase, we applied the AICM module algorithm on the constructed simulation data set. The correlation result is presented in Table 5. We select the false-positive rate (FPR) and the true-positive rate (TPR) as the correlation effect measurement parameters. The parameters involved in our experiments are {P, N, TP, FP}, which present the quantity of APT alerts, the quantity of random isolated alerts, the quantity of true-positive APT alerts, and the quantity of false-positive APT alerts, respectively. Then, the correlation effect measurement parameters TPR and FPR can be formalized as follows:

(25) $$\mathrm{TPR}=\frac{\mathrm{TP}}{\mathrm{TP}+\mathrm{FN}}$$

(26) $$\mathrm{FPR}=\frac{\mathrm{FP}}{\mathrm{FP}+\mathrm{TN}}$$

**Figure 10:**
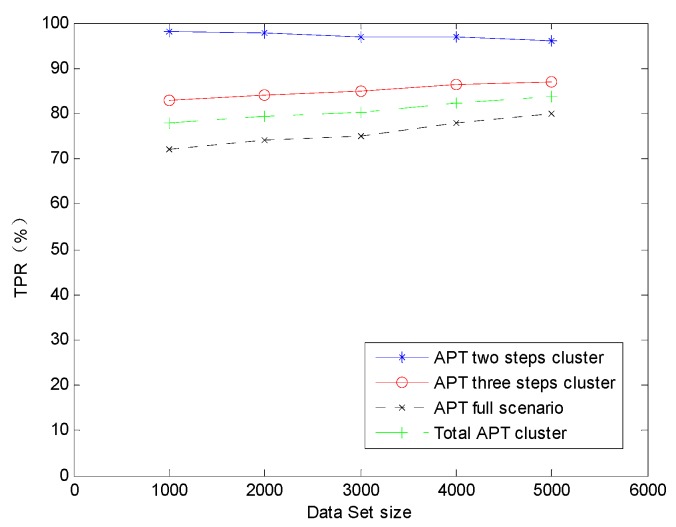
The TPR varies with data set size changes.

**Figure 11:**
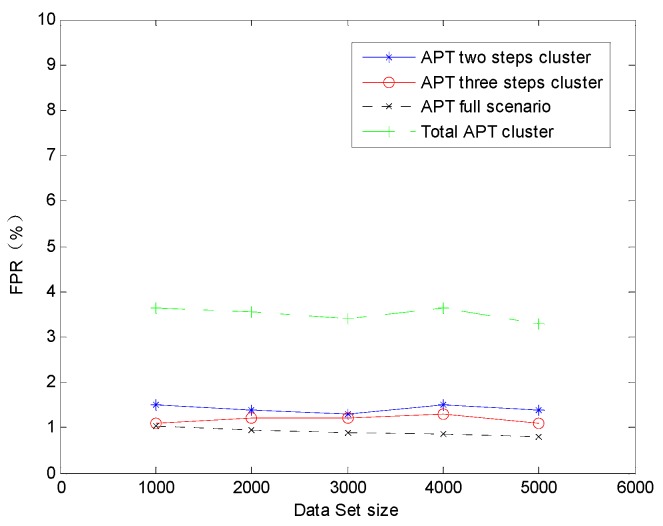
The false-positive rate (FPR) varies with the data set size changes.

Here, we can find the TPR of the two steps *Cluster_sub_* is higher than any other clusters. It is evident that the TPR is lower with the alert instance cluster steps quantity increaser. This is primarily because more alert instances correlation process will increase the possibility of the random isolated alert instances to be unexpected correlated. When decreasing the TPR, the unexpected random isolated alert instances correlation can also incur the false positive correlation result. As the larger step quantity of the clusters, the stronger ability they equipped to amend the previous correlation by the later alert instances, so that FPR is lower with the alert instance cluster steps quantity increaser. In general, holistic TPR and FPR are well satisfied. To reveal the effect on the detection accuracy with the data set size changing we also applied the AICM module algorithm on the sectional simulation data sets whose alert quantity are 1000, 2000, 3000 and 4000. We can get the TPR variation trend on the condition of data sets size changing in Figure 10. It is obvious that TPRs of APT three steps cluster, APT full scenario and Total APT cluster increase with the data set size expanding. This phenomenon is due to more alerts can also enhance the amendment ability of the pre-step correlation operation. However, TPR of the APT two step cluster slightly decreases with the data set size expanding, because of more alerts within two certain steps only can increase the risk of leaving out some correlations and there are no pre-step correlation operations to be amended. However, we cannot dig out any variation trends of FPR on the condition of data sets size changing in Figure 11, and it is probably due to the absolute value of the FPRs are so low. 

#### 5.1.3. Performance Comparison Between AICM And Existing APT Detection Systems

We compared the performance of the developed AICM module with the three typical APT scenarios detection methods by operating the three methods on the simulation data set we generated in Section 5.1.1 to show the advantage of ACIM. We also choose the false-positive rate (FPR) and the true-positive rate (TPR) as the correlation effect measurement parameters. The value of TPR and FPR can be calculated according to Equations (25) and (26). The FPR and TPR of each method can be regarded as the comparative results which are presented in Table 6.

We can get the evident results that the other three proposed APT scenarios detection methods are not able to handle the problem of balancing the higher TPR and lower FPR properly. It is obvious to see that AICM acquire satisfactory TPR with not too high FPR. As the only method can approximately get the similar performance to AICM, the C&C-based method has disadvantages of failing to accomplish real-time APT attack detection. Comparing the experiment results of Spear phishing based and TerminAPTor, we find under the premise of similar valid detection capability of APT attack (The TPR values of the two methods are approximately equal), increasing the step quantity will incur more false positive APT alerts. 

### 5.2. Evaluation of the Log Instance Correlation module

We implement the algorithm of LICM in Python and make full use of the convenience of package python-Louvain to accomplish log instance community detection. The experiment environment is described as follows: (1) a victim Windows 10 64-bit operating system running on a host with an Intel Core i5-7200u 2.0 GHz CPU, 8GB RAM. (2) an attack Windows 10 64-bit operating system running on a host with an Intel Core i7-8550u 2.53 GHz CPU, 16GB RAM. We also assign extra roles for the attack host: FTP server, C&C server, and Apache server.

#### 5.2.1. Data Generation

To construct the log data set and evaluate the LICM module algorithm we record the log data from the log providers and the log data quantity of each provider is shown in Table 7. We record the log data by routinely work without perceiving that some operations have triggered APT attack activities. We obtain these log data after some attacks have launched to simulate the APT attack scenario.

#### 5.2.2. Correlation Performance

**Figure 12:**
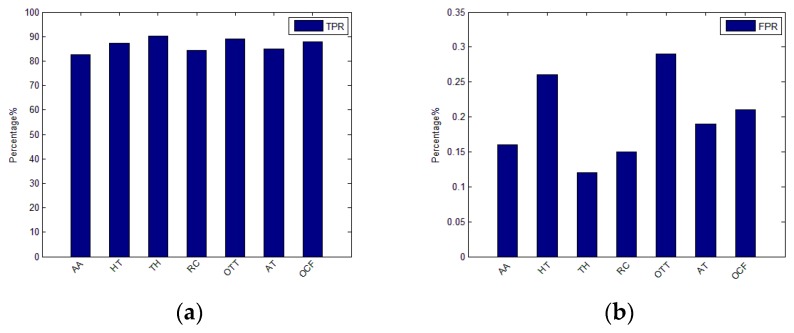
Evaluation LICM module detection result; (**a**) true-positive rate (TPR); (**b**) FPR.

We have applied the LICM module algorithm on the recorded log dataset to evaluate the performance of log community detection on 7 APT attack scenarios such as Attack on Aerospace (AA), Hacking Team (HT), Tibetan and HK (TH), Russian Campaign (RC), Op-Tropic Trooper (OTT), APT on Taiwan (AT), and Op-Clandestine Fox (OCF). We also select the FPR and TPR as the correlation effect measurement parameters. Log instances within any detected community are regarded as malicious ones and the others as benign ones. The TRP is the portion of the malicious log instance and benign log instance which have been correctly classified. The FPR is the portion of the actual benign log instances, which are unexpectedly classified into the malicious cluster. The results are shown in Figure 12; we can see that TPRs of LICM module work well on the 7 typical APT scenarios and the FPRs are also medium.

### 5.3. Attack Scenario Reconstruction Time

**Figure 13:**
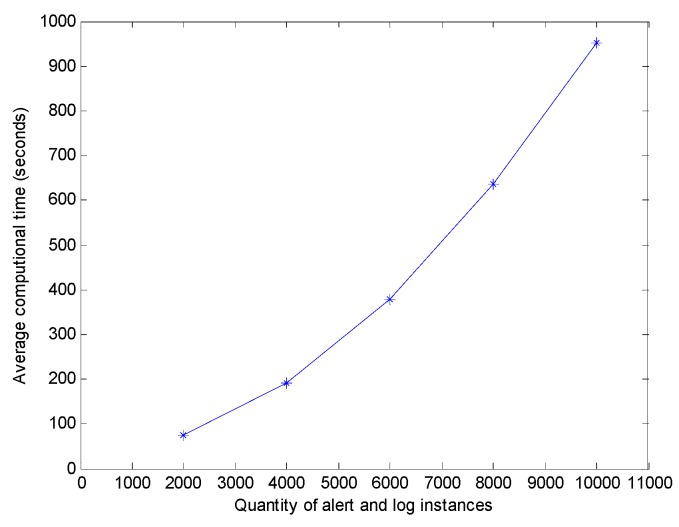
Computational time varies with the instance quantity changes.

The time complexity of the attack scenario reconstruction versus the number of alert and log instances is one of the most important parameters in evaluating the proposed technique. The scenario reconstruction time is the average time from the time an alert is generated to the time this newly generated alert is correlated to one of the attack scenarios plus the average time of the log associated with that newly generated alert to be classified to a certain cluster. Figure 13 depicts the impact of the number of alert and log instances on the average reconstruction time, which smoothly increases as the number of instances increases.

## 6. Conclusions

In this paper, we proposed the APT alerts and logs correlation method to accomplish the cyber situation comprehension in IoT systems. To appropriately reduce the communication overhead of the proposed method, we provide a framework of deploying APTALCM on the edge computing-based IoT system. To recognize attack intentions, a similarity measures method based on SimRank is provided. We also proposed an APT alert instances correlation method to reconstruct APT attack scenarios and an APT log instances correlation method to detect log instance communities. The experimental results demonstrate that the APTALCM has higher TPR with acceptable FPR.

## Figures and Tables

**Table 1:** The attributes of Advanced Persistent Threat (APT) alert class.

Number	Attributes	Description
1	Timestamp	The time of the alert occurs
2	Alert_Type	The type of alert
3	Src_Ip	The source IP of the attack step
4	Dest_Ip	The destination IP of the attack step
5	Src_Port	The source port number of the attack step
6	Dest_Port	The destination port number of the attack step
7	Victim_HostIp	The IP of the host which victimized by the attack step

**Table 2:** Logs used for cyber situation perception comprehension.

Number	Logs	Providers
1	HTTP	Internet explorer
2	Object access	Audit
3	DNS	Tshark
4	Authentication	Syslogd
5	Process create	Audit
6	WFP connect	Audit

**Table 3:** The attributes extracted from log data.

Number	Logs	Attribute	Description
1	Log1-Log6	timestamp	Event timestamp
2	Log3	q_domain	DNS queried domain name
3	Log3	r_ip	DNS resolved IP address
4	Log2 Log5 Log6	pid	base-16 process ID
5	Log5	ppid	base-16 parent process ID
6	Log2 Log4 Log5 Log6	pname	process
7	Log6	h_ip	host IP address
8	Log6	h_port	host port number
9	Log6	d_port	destination port number
10	Log6	d_ip	destination IP address
11	Log6	type	request/response
12	Log1	get_q	absolute path of GET
13	Log1	post_q	absolute path of POST
14	Log1	res_code	response code
15	Log1	h_domain	host domain name
16	Log1	referer	refer of requested URI
17	Log1	res_loc	location to redirect
18	Log2	acct	principle of this access
19	Log2	objname	object name

**Table 4:** The matchup between APT attack scenario steps and alert instances.

Step Number	APT Step	Alerts Instance
Step 2	(P) Point of entry	$I\left(p_{1}\right)$Domain_instance
		$I\left(p_{2}\right)$Disguised_exe_instance
		$I\left(p_{3}\right)$Hash_instance
Step 3	(C) C&C communication	$I\left(c_{1}\right)$Domain_flux_instance
		$I\left(c_{2}\right)$Ip_instance
		$I\left(c_{3}\right)$Ssl_instance
Step 5	(A) Asset/Data discovery	I(*a*_1_)Scan_instance
Step 6	(D) Data exfiltration	I(*d*_1_)Tor_intance

**Table 5:** Evaluation AICM module correlation result.

APT Attack Cluster	Correlated Quantity	FP	TP	FN	TN	N	P	FPR	TPR
APT two steps cluster	2*83	2*35	2*48	4	4830	4900	100	1.4%	96%
APT three steps cluster	3*106	3*19	3*87	39	4643	4700	300	1.2%	87%
APT full scenario	4*121	4*10	4*120	120	4360	4400	600	0.9%	80%
Total APT cluster	968	167	837	163	3833	4000	1000	4.2%	83.7%

**Table 6:** The comparative results between AICM and other APT scenarios reconstruction method.

APT Scenarios Reconstruct Method	Efficiency	Step Quantity	FPR	TPR
AICM	Real-time	Four steps	4.2%	83.7%
Spear phishing based	Real-time	One step	15.9%	94.3%
TerminAPTor	Real-time	Four steps	25.64%	98.8%
C&C-based	Off-line	One step	1.2%	79.6%

**Table 7:** APT log instance size.

Log	Quantity	Size (KB)
HTTP	3345	16,530
Object access	282	5789
Process create	261	6420
DNS	510	72
WFP	734	18,453
